# *De novo* genome assembly of *Akanthomyces muscarius*, a biocontrol agent of insect agricultural pests

**DOI:** 10.1099/acmi.0.000568.v3

**Published:** 2023-06-12

**Authors:** Zoltan Erdos, David John Studholme, Ben Raymond, Manmohan D. Sharma

**Affiliations:** ^1^​ Ecology and Conservation, University of Exeter, Penryn, TR9 10FE, UK; ^2^​ Biosciences, University of Exeter, Exeter, EX4 4QD, UK

**Keywords:** entomopathogenic fungi, genome assembly, biological control, hypocrealen fungi

## Abstract

The entomopathogenic fungus *Akanthomyces muscarius* is commonly used in agriculture to manage insect pests. Besides its use as a commercially important biological control agent, it also presents a potential model for studying host–pathogen interactions and the evolution of virulence in a laboratory setting. Here, we describe the first high-quality genome sequence for *A. muscarius*. We used long- and short-read sequencing to assemble a sequence of 36.1 Mb with an N_50_ of 4.9 Mb. Genome annotation predicted 12347 genes, with 96.6 % completeness based on the core Hypocrealen gene set. The high-quality assembly and annotation of *A. muscarius* presented in this study provides an essential tool for future research on this commercially important species.

## Data Summary

The genome assembly and annotation have been deposited at the GenBank database under accession number JAJHUN000000000. The version described in this paper is version JAJHUN010000000. The raw sequence data can be found under BioProject accession PRJNA777543. The code used to obtain results can be found in the Supplementary material.

## Introduction

Entomopathogenic fungi (EPF) are diverse, naturally occurring pathogens of arthropods. Their potential as biological control agents has prompted substantial research effort in collection, description and fundamental biology [1–5]. EPF have been studied as candidate control agents against sucking pests, such as aphids, which are hard to control with bacterial or viral microbial biocontrol agents (MCAs) that need to be ingested [6]. The most well-studied group of EPF are the asexual forms of ascomycetes, due to the relative ease of *in vitro* mass-propagation of infective spores that can be formulated and applied as sprays [7, 8]. The majority of these belong to *Beauveria* spp. and *Metarhizium* spp., many of which have been developed for inundative control programmes against a wide range of insect pests [9, 10].

*Akanthomyces muscarius* is an EPF belonging to the order *Hypocreales*, with activity against a wide range of economically important invertebrate crop pests [11]. It has been commercialized as a biological control agent and used against whiteflies and aphids in greenhouses. This species is widely distributed, with a preference for temperate climates. Besides being an important resource for pest management, it also offers a model for studying host–pathogen interactions in a laboratory settings. The availability of genomic resources for this fungus will aid in strain improvement and innovation in biocontrol and the presented draft genome assembly and annotation therefore, offers an important resource for current and future research using this entomopathogen. Furthermore, we compare our genome assembly to that of other entomopathogenic fungi belonging to the family *Cordycipitaceae*.

## Methods

### DNA and RNA extraction and library preparation

*A. muscarius* mycelia were harvested from 14-day-old fungal cultures grown on Sabouraud dextrose agar (SDA) (Thermo Scientific, UK) at 22±1 °C in the dark using a sterile scalpel. High-molecular-weight DNA was extracted from fungi using the Genomic-tip 100/G kit (Qiagen, UK). Harvested mycelia were homogenized in liquid nitrogen before proceeding with DNA extraction following manufacturer’s instructions for tissue extraction. The only modification to the recommended protocol was a prolonged lysis step overnight and an additional centrifugation after lysis (13000g for 6 min). The extracted DNA was quantified using a NanoDrop spectrophotometer 2000 (Thermo Scientific, UK) and Qubit Fluorometer using the Qubit dsDNA BR assay kit (Thermo Scientific, UK). DNA integrity was checked using agarose gel electrophoresis. Long-read Pacific Biosciences sequencing was performed using a single PacBio Sequel SMRT cell. Additionally, we generated an Illumina library using the Illumina NEBNext Ultra II FS Kit, that was sequenced on a single lane of Illumina NovaSeq SP.

Total RNA was extracted from both fungal mycelia cultured on SDA medium and infected *Myzus persicae* (one day after host death) using the ISOLATE II RNA Mini Kit (Bioline, UK) following manufacturers’ instructions for cells and tissue. Four biological replicates were used for both *in vitro* and *in vivo* extraction. Twenty aphid cadavers were pooled for each replicate of *in vivo* RNA extraction. The integrity of RNA was determined by agarose gel electrophoresis, quantified using a NanoDrop spectrophotometer 2000 and Qubit Fluorometer using the Qubit RNA BR assay kit (Thermo Scientific, UK). RNA-seq libraries were prepared using RNA as template following polyA enrichment and sequenced on Illumina NovaSeq platform with 150 bp paired end (PE) read metric to > 68M PE reads per replicate (Novogene, UK). Read quality was assessed using FastQC v0.11.8 [12], and low-quality reads and adapter sequences were removed using TrimGalore v0.6.5 [13].

### Genome assembly

Cutadapt v1.2.1 [14] was used to trim the Illumina genomic reads from adapter sequences with the flag – O 3, trimming any sequence that matched the adapter sequence for 3 bp or more. An additional trim was performed using Sickle v1.200 [15] with a minimum window quality score of 20. Reads shorter than 15 bp in length after trimming were removed.

In order to find an optimal hybrid assembly strategy, we evaluated two pipelines, MaSuRCA v4.0.5 [16] and WENGAN v0.2 [17]. The MaSuRCA workflow offers three different assemblers: CABOG [18], SOAPdenovo2 [19] and Flye [20]. The pipeline was tested using CABOG assembler, designed for long-read assembly. The WENGAN pipeline was used in WENGAN-D setting, based on the DISCOVARdenovo short-read assembler [21]. Additionally, we performed a HASLR v0.8a1 [22] assembly. Furthermore, we tested a Flye-only assembly, using the latest version of Flye v2.9-b1768 [20] in both reduced coverage for initial disjointing assembly (-asm 50) and uneven coverage mode (-meta).

All parameters were set as default, with the exception of genome size of 36 Mb for the Flye assemblies. The resulting Flye assemblies were subjected to polishing with PacBio reads using Racon v1.4.20 with one to four iterations [23]. This was followed by an additional correction step with Illumina reads using POLCA (built-in MaSuRCA v4.0.5 polisher) [16]. Read mapping to the genome assembly was performed with minimap2 v2.22 [24] for PacBio and BWA v2.2.1 [25] for Illumina reads.

Basic metrics for *A. muscarius* genome assemblies were calculated using QUAST v5.1.0rc1 [26]. Genome completeness was assessed using the BUSCO v5.2.2 (Benchmarking Universal Single-CopyOrthologues) pipeline with the *Hypocreales* odb10 dataset consisting of a total of 4494 BUSCOs [27]. Repetitive elements were identified and masked in the genome assemblies using RepeatMasker v4.0.9 [28]. The best assembly was selected for *ab initio* annotation using the BRAKER2 v2.1.6 [29] pipeline based on gene predictions from GeneMark-ES v4.68 [30] and AUGUSTUS v3.3.0 [31] using the created RNA-seq libraries as evidence. Transcriptome data mapping against the repeat-masked genome was performed using HISAT2 v2.1.0 [32], and sorting and indexing were carried out with SAMtools v1.10 [33]. BRAKER2 was run with the flags *–softmasking*—*gff3*. The resulting BAM files from strand-specific RNA-seq alignments were split into forward and reverse strands when parsed to BRAKER2 in order to improve accuracy, as recommended in the BRAKER2 documentation. Chromosome completeness was estimated by searching for the TTAGGG/CCTAAA telomere sequence commonly found in eukaryotes using Telomere Identification toolkit (tidk) v0.2.31 (https://github.com/tolkit/telomeric-identifier).

## Results

### Library preparation and genome features

Long-read sequencing using Pacific Biosciences yielded 5.9 million reads with an N_50_ of 26 kb. Illumina sequencing resulted in 44.5 million pairs of 150 bp with an average insert size of 210 bp.

Both Flye with –asm option and MaSurCA assembled 12 contigs with an N_50_ of 4.8 Mb and 4.7 Mb, respectively. WENGAN produced 16 contigs with a higher N_50_ of 5.3 Mb, while HASLR assembled 101 contigs (Table 1). All assembly methods produced assemblies with sizes in the range of 35.4–36.3 Mb. Based on the assembly statistics and its higher average coverage, we proceeded with the Flye_asm assembly. Polishing the Flye_asm assembly with short-read data using Racon (1-4 iterations) or POLCA did not significantly improve the assembly statistics. However, based on the single copy BUSCO scores, we chose the POLCA-corrected Flye_asm assembly for annotations. This final assembly had a total length of 36.2 Mb, N_50_ of 4.86 Mb and a G+C content of 53% (Table 1). This is consistent with the 30–40 Mb genome sizes reported for most assemblies of filamentous ascomycete fungi, such as the close relative *Akanthomyces lecanii* (35.59 Mb genome size, 53.1% GC content) [34]. Detailed assembly properties are given in Table 1. Analysis of BUSCOs revealed a high degree of completeness, with 96.6 % of the Hypocreales test gene set found to be present (combined single copy and duplicate BUSCOs, Table 2). Search for telomere sequences (TTAGGG) suggests that five contigs represent full length chromosomes, while other contigs contain only partial telomere sequences. This should be further investigated to fully resolve chromosomes (Fig. S1).

**Table 1.** Summary statistics of genomes obtained with different assemblers

	MaSurCA	Flye-asm	Flye-meta	FlyePOLCA	HASLR	WENGAN
No. of contigs	12	12	24	**12**	101	16
Largest contig (Mb)	7.41	7.45	7.41	**7.45**	6.01	7.39
Total length (Mb)	36.08	36.21	36.1	**36.1**	35.53	35.62
GC (%)	53.02	53.01	53.01	**53.01**	53.20	53.03
N_50_	4.7	4.86	5.36	**4.86**	1.54	5.34
N_90_	2.32	2.34	2.32	**2.34**	2.71	3.83
L_50_	3	4	3	**4**	6	3
L_90_	7	7	7	**7**	25	7
Avg. coverage depth	875	893	891	**893**	874	865
Complete single-copy BUSCOs	4317 (96.1 %)	4316 (96.0 %)	4317 (96.1)	**4318** (96.1 %)	4203 (93.5 %)	4317 (96.1 %)

**Table 2.** Comparison of genomes of other insect pathogenic fungi from the order *Hypocrelaes*, obtained from GenBank

	*Akanthomyces lecani*	*Beauveria bassiana*	*Cordyceps militaris*	*Akanthomyces muscarius*
GenBank accession	GCA_001636795.1	GCA_014607475.1	GCA_008080495.1	GCA_028009165.1
No. of contigs	197	12	7	12
Total length (Mb)	35.60	37.13	33.62	36.21
GC (%)	53.02	48.91	50.91	53.01
N_50_	3.95	4.61	5.78	4.86
BUSCO (single-copy and duplicate, %)	96.6	96.7	95.7	96.6
Total repeat content (%)	2.97	6.99	3.03	5.22

A total of 5.2% of bases were identified as repetitive elements by RepeatMasker. That is comparable to other fungi within *Cordycipitaceae* (Table 2). These repeats included long interspersed nuclear elements (LINEs), long terminal repeat (LTR) retrotransposons and other DNA elements (Table 3). The BRAKER2 pipeline incorporating the RNA-seq data predicted 12 347 genes and 12 961 coding DNA sequences (CDS) in the *A. muscarius* genome.

**Table 3.** Repeat content of *A. muscarius* identified by RepeatMasker

Class	Count	Bp masked	% masked
**DNA**	83	97 673	0.27
CMC-EnSpm	7	1656	0.00
MULE-MuDR	9	32 509	0.09
PiggyBac	19	18 183	0.05
TcMar-Fot1	142	161 249	0.45
**LINE**			
CRE	41	243 538	0.67
Tad1	201	235 585	0.65
**LTR**			
Gypsy	195	385 389	1.06
**Unknown**	774	348 153	0.96
Total interspersed	1471	1 523 935	4.21
Low complexity	719	33 816	0.09
Satellite	46	5251	0.01
Simple repeat	7517	301 711	0.83
rRNA	15	24 436	0.07
**Total**	9768	1 889 149	5.22

## Conclusions

Currently there are 69 genomes available within the family *Cordycipitaceae* with *Beauveria* and *Cordyceps* being the most well represented insect parasitic fungi with 32 and 16 genomes, respectively (NCBI). *Akanthomyces*, a commercially relevant family of insect pathogen fungi is only represented by three genome assemblies for the species *A. lecanii.* The near-chromosome-level genome assembly for *A. muscarius* presented here is currently the best available assembly in the genus *Akanthomyces.* It will provide an important resource for studying host-pathogen interactions with this fungus as well as strain improvement for biological control.

## Supplementary Data

Supplementary material 1
