# Supplementary material for: De novo genome assembly of Akanthomyces muscarius, a biocontrol agent of insect agricultural pests
Source: Access Microbiol. 2023 Jun 12;5(6):acmi000568.v3. doi: 10.1099/acmi.0.000568.v3 (PMC10323777; doi:10.1099/acmi.0.000568.v3)
Supplement: Supplementary material 1 [file acmi-5-568.v3-s001.pdf]

Used modules:

Flye/2.9-b1768

haslr/0.8a1

wengan/v0.2

MaSuRCA/4.0.5

minimap2/2.22

Racon/1.4.20

Quast/v5.1.0rc1

RepeatMasker/v4.0.9-p2

Braker 2.1.6, BUSCO v5.2.2.

tidk 0.2.31

Flye-asm

flye --pacbio-hifi "${file_dir}/m64147e_210730_142608.hifi_reads.fastq.gz" --out-dir "${out_dir}/flye-asm" --threads 95 --asm-coverage 50 --genome-size 36m

Flye-meta

flye --pacbio-hifi "${file_dir}/m64147e_210730_142608.hifi_reads.fastq.gz" --out-dir "${out_dir}/flye-meta" --threads 95 --meta --genome-size 36m

HASLR

haslr.py -t 60 -o amusc_haslr -g 36m -l
01_pacbio_hifi_fastq/m64147e_210730_142608.hifi_reads.fastq -x pacbio -s
01_illumina/Trimmed/Sample_14-ANC/14-ANC _L002_R1_001.fastq.gz
01_illumina/Trimmed/Sample_14-ANC/14-ANC_ L002_R2_001.fastq.gz

MaSurCA

masurca -i ${illumina_dir}/14-ANC_L002_R1_001.fastq.gz,${illumina_dir}/14-ANC _L002_R2_001.fastq.gz -r ${pacbio_dir}/m64147e_210730_142608.hifi_reads.fastq.gz -t 95 --output ${out_dir}/

Wengan

wengan.pl -t 90 -p amusc_wengan -g 36 -x pacraw -a A -l 01_pacbio_hifi_fastq/m64147e_210730_142608.hifi_reads.fastq.gz -s 01_illumina/Trimmed/Sample_14-ANC/14-ANC _L002_R1_001.fastq.gz, 01_illumina/Trimmed/Sample_14-ANC/14-ANC _L002_R2_001.fastq.gz

Racon polish

```
cd $WORKSPACE/03_flye/flye-asm
base=$WORKSPACE/03_flye/flye-asm
genome=$base/assembly.fasta
pacbio_r=01_pacbio_hifi_fastq/m64147e_210730_142608.hifi_reads.fastq.gz
run1=$base/Polish_minimap_old/racon/run1
run2=$base/Polish_minimap_old/racon/run2
run3=$base/Polish_minimap_old/racon/run3
run4=$base/Polish_minimap_old/racon/run4
out="racon"

echo "making directories"
mkdir -p $run1
mkdir -p $run2
mkdir -p $run3
mkdir -p $run4

minimap2 -t 64 -ax map-pb $genome $pacbio_r -o $run1/aligned.sam
racon -t 64 $pacbio_r $run1/aligned.sam $genome > $run1/${out}_1_polished.fasta
##
minimap2 -t 64 -ax map-pb $run1/${out}_1_polished.fasta $pacbio_r -o $run2/aligned.sam
racon -t 64 $pacbio_r $run2/aligned.sam $run1/${out}_1_polished.fasta > $run2/${out}_2_polished.fasta
```

##

minimap2 -t 64 -ax map-pb $run2/${out}_2_polished.fasta $pacbio_r -o $run3/aligned.sam

racon -t 64 $pacbio_r $run3/aligned.sam $run2/${out}_2_polished.fasta > $run3/${out}_3_polished.fasta

##

minimap2 -t 64 -ax map-pb $run3/${out}_3_polished.fasta $pacbio_r -o $run4/aligned.sam

racon -t 64 $pacbio_r $run4/aligned.sam $run3/${out}_3_polished.fasta > $run4/${out}_4_polished.fasta

##

POLCA polish

polca.sh -a 03_flye/flye-asm/assembly.fasta -r "${illumina_dir}/14-ANC _L002_R1_001.fastq.gz ${illumina_dir}/14-ANC_ L002_R2_001.fastq.gz" -t 60

Quast

ass_dir=$WORKSPACE/03_flye/flye-asm/Polish_minimap_old

seq_dir=$WORKSPACE/01_pacbio_hifi_fastq

seq_dir2=$WORKSPACE/03_flye/flye-asm

seq_dir3=$WORKSPACE/03_flye/flye-meta

seq_dir4=$WORKSPACE/03_flye/flye-asm/POLCA_polish

seq_dir5=$WORKSPACE/03_HASLR/amusc_haslr/asm_contigs_k49_a3_lr25x_b500_s3_sim0.85

seq_dir6=$WORKSPACE/03_wengan

seq_dir7=$WORKSPACE/03_MaSurCA/CA.mr.95.17.15.0.02

quast --threads 43 --fungus --gene-finding --conserved-genes-finding --pacbio ${seq_dir}/m64147e_210730_142608.hifi_reads.fastq.gz -L -o ${qdir}/ ${seq_dir7}/primary.genome.scf.fasta ${ass_dir}/racon_1_polished.fasta ${ass_dir}/racon_2_polished.fasta ${ass_dir}/racon_3_polished.fasta ${ass_dir}/racon_4_polished.fasta ${seq_dir2}/assembly.fasta ${seq_dir3}/assembly.fasta ${seq_dir4}/assembly.fasta.PolcaCorrected.fa ${seq_dir5}/asm.final.fa ${seq_dir6}/amusc_wengan.SPolished.asm.wengan.fasta

BUSCO

busco -c 80 -f -m genome -i ${seq_dir2}/assembly.fasta -o flye-asm -l hypocreales_odb10

busco -c 80 -f -m genome -i ${seq_dir7}/primary.genome.scf.fasta -o MaSurCA -l hypocreales_odb10

busco -c 80 -f -m genome -i ${seq_dir5}/asm.final.fa -o HASLR -l hypocreales_odb10

busco -c 80 -f -m genome -i ${seq_dir3}/assembly.fasta -o flye-meta -l hypocreales_odb10

busco -c 80 -f -m genome -i ${seq_dir4}/assembly.fasta.PolcaCorrected.fa -o FlyePolca -l hypocreales_odb10

busco -c 80 -f -m genome -i ${seq_dir6}/amusc_wengan.SPolished.asm.wengan.fasta -o wengan -l hypocreales_odb10

RepeatMasker

RepeatMasker -e ncbi -gff -lib 05_RepeatMasker/consensi_Toby_library.fa.classified -pa 40 03_flye/flye-asm/POLCA_polish/assembly.fasta.PolcaCorrected.fa –xsmall

Braker

braker.pl --cores 32 --useexisting --fungus --gff3 --workingdir=$wd --softmasking --ALIGNMENT_TOOL_PATH=/cm/shared/admin-apps/GeneMark-ES/4.68_lic/ProtHint/bin --etpmode --genome=$refgenome --species=akanthomyces_muscarius --bam=$A1forward,$A1reverse,$A2forward,$A2reverse,$A3forward,$A3reverse,$A4forward,$A4reverse,$A5forward,$A5reverse,$A6forward,$A6reverse,$A7forward,$A7reverse,$A8forward,$A8reverse --stranded=+,-,+,-,+,-,+,-,+,-,+,-,+,-,+,- --prot_seq=$transcript_prot

tidk

tidk search --string TTAGGG -o unmaskedTTAGGG --dir ttaggg_repeats/ --extension tsv seq_dir4

tidk plot --tsv unmaskedTTAGGG_telomeric_repeat_windows.tsv"

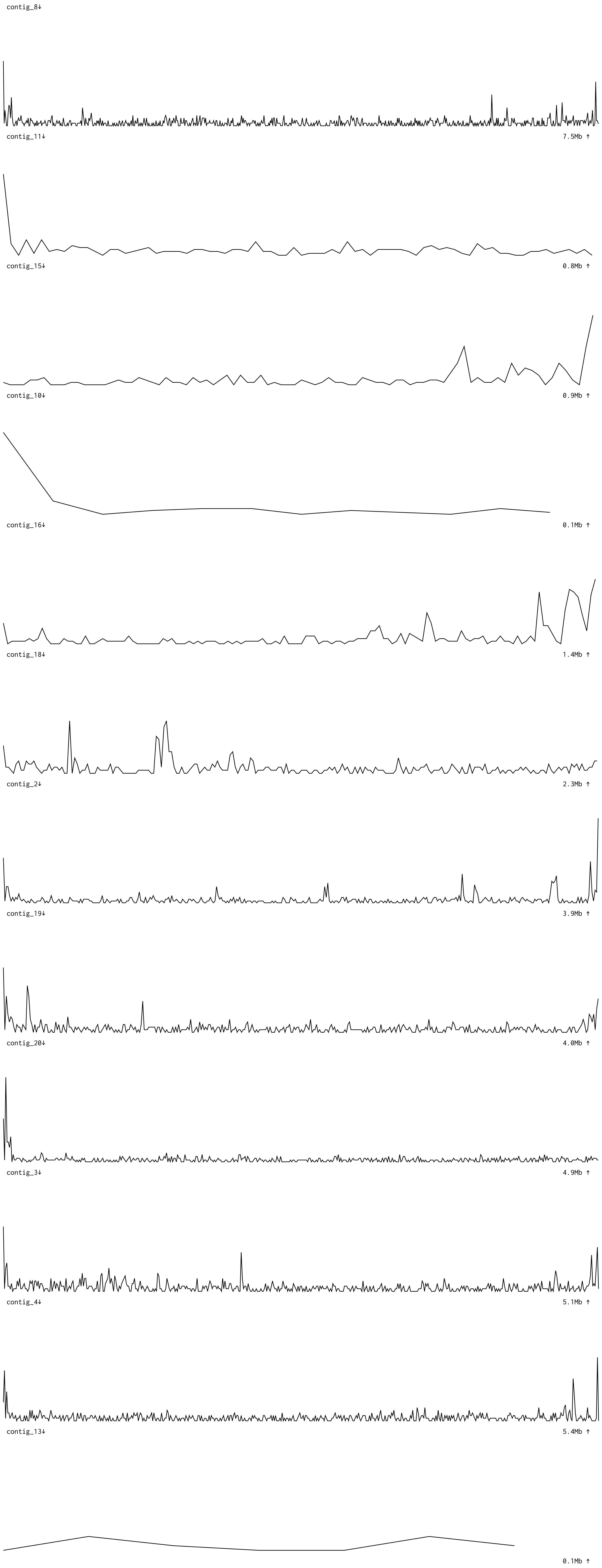

S1. Telomere repeat motif TTAGGG counts along the assembled contigs (TIDK v0.2.31)
